# A rapidly developing outbreak of clade Ib mpox among gay, bisexual and other men who have sex with men associated with severe proctitis, Northern Ireland, June 2026

**DOI:** 10.2807/1560-7917.ES.2026.31.30.2600604

**Published:** 2026-07-30

**Authors:** Abbie Harrison, Catherine Burns, Corey Magee, Monica Sloan, Joy Murphy, Laura McCartney, Maeve Middleton, Emma McCarty, Eoin Walker, John White, Melissa Perry, Nuala Cavanagh, Bill Gibson, Stephen Megarity, Kathy Li, Susan Feeney, Declan Bradley, Peter Naughton, Rachel Coyle

**Affiliations:** 1 Public Health Agency, Belfast, United Kingdom; 2 Belfast Health and Social Care Trust, Northern Ireland, United Kingdom; 3 Northern Health and Social Care Trust, Northern Ireland, United Kingdom; 4 Western Health and Social Care Trust, Northern Ireland, United Kingdom; 5 South Eastern Health and Social Care Trust, Northern Ireland, United Kingdom; 6 Southern Health and Social Care Trust, Northern Ireland, United Kingdom; * These authors contributed equally to this work and share first authorship.

**Keywords:** mpox, MPXV, outbreak, public health surveillance, sexual health, men who have sex with men (MSM), vaccination

## Abstract

We describe a rapidly expanding clade Ib mpox outbreak among gay, bisexual and other men who have sex with men in Northern Ireland. Following 12 months with no detected mpox cases, 22 confirmed cases were notified between 6 and 30 June 2026. Cases had a median age of 41 years; 19 had not travelled outside the United Kingdom or Ireland in the 21 days before symptom onset. The outbreak is characterised by severe anorectal disease and presentation to non-sexual health settings, including secondary care.

Clade Ib mpox has been reported in Europe since late 2024, with autochthonous transmission since late 2025 [1-3]. Before June 2026, no clade Ib mpox had been detected in Northern Ireland, and clade IIb had not been identified for over 12 months. We describe an ongoing, rapidly expanding large clade Ib mpox outbreak comprising 22 cases among gay, bisexual and other men who have sex with men which began in early June 2026. 

## Outbreak description

On 6 June 2026, the first three laboratory-confirmed clade Ib mpox cases in Northern Ireland were notified to the Public Health Agency (PHA), which is the authority with responsibility for health protection in Northern Ireland. All three cases were adult males who had had at least one new sexual contact in the 21 days before symptom onset. No case reported known contact with a person with suspected or confirmed mpox.

By 2 July 2026, 22 laboratory-confirmed cases had been notified to the PHA. Earliest symptom onset ranged from 27 May to 24 June 2026, and specimen dates ranged from 5 June to 26 June 2026 (Figure). All cases were male, with a median age of 41 years (mean: 42; interquartile range (IQR): 33–52) at notification. The majority of cases were classified as gay, bisexual and other men who have sex with men (gbMSM) based on self-reported sexual behaviour and/or orientation. Nineteen cases reported no international travel outside of the United Kingdom (UK) or Ireland in the 21 days before the date of onset. Mpox vaccination status was known for 18 cases: six reported prior vaccination with at least one dose, while 12 cases reported no prior vaccination.

**Figure:**
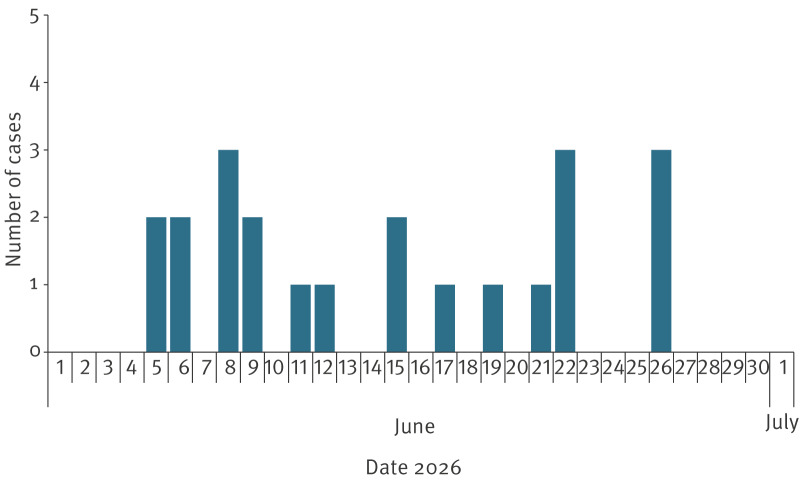
Laboratory-confirmed mpox clade Ib cases, by specimen date, Northern Ireland, 26 May–1 July 2026 (n = 22)

### Clinical characteristics

All cases were symptomatic: 20 of the 22 cases reported prodromal symptoms, 20 cases developed a mucocutaneous rash or lesions, and nine reported anorectal symptoms, including proctitis or perianal abscess.

Among the 22 cases, 12 cases initially presented to Sexual Health and HIV (SHH) services, while 10 cases initially presented to emergency departments or out-of-hours primary care. Mpox testing was not performed at the initial presentation in eight of these 10 patients, six of whom were subsequently tested by SHH services. The median time from first presentation to diagnostic swabbing was 1.5 days (range: 0–9) among those initially presenting to emergency or general healthcare settings, compared with 0 days (range: 0–8) among those who presented directly to SHH.

Five cases required mpox-related hospital admission (one case to intensive care, one case to surgical care and three cases to medical care). The characteristics of the hospitalised cases are summarised in the Table. Hospitalised cases had a median age of 40 years (mean: 39 years; IQR: 34–46). One case had a co-infection with herpes simplex virus identified during their illness episode. All five hospitalised cases reported prodromal symptoms, three of five reported mucocutaneous rash or lesions, and four of five reported anorectal symptoms. Before mpox was considered as the underlying aetiological factor for these presentations, one case received surgical intervention, one case was scheduled for examination under anaesthesia and computed tomography imaging demonstrated severe proctitis in a third case.

**Table:** Characteristics of individuals with clade Ib mpox infections who were hospitalised in Northern Ireland, June 2026 (n = 5)

Case	Time to diagnostic test ^a^ (days)	Clinical description	Co-infection ^b^	Previous mpox vaccination
1	0	Generalised rash, lesions, febrile prodrome; admitted for pain management	Yes	Unknown
2	3	Peri-anal abscess, localised rash (finger)	No	Unknown
3	9	Fever, abdominal pain, proctitis, no rash	No	Yes (3 doses)
4	0	Admitted with suspected IBS, proctitis, septic shock, no widespread skin lesions	No	No
5	2	Proctitis, penile ulcer	No	No

IBS: irritable bowel syndrome.

^a^ Time from first healthcare attendance to diagnostic swab taken (as proxy for mpox first considered in differential diagnosis).

^b^ Co-infection present (i.e. another known positive result for another infection during the mpox episode), excluding individuals diagnosed with HIV with undetectable viral load and no evidence of immunosuppression. Additional diagnostic testing may include screening for sexually transmitted infections (chlamydia, gonorrhoea, syphilis), blood-borne viruses, herpes simplex virus and varicella zoster virus, however, not all cases had full sexually transmitted infections and syndromic testing.

Diagnostic uncertainty was also reported for some cases who were not hospitalised but had presented for care in SHH clinics. One case was empirically treated for primary syphilis and subsequently diagnosed with mpox 3 days after first healthcare attendance. Another case was provisionally diagnosed and treated for peri-anal herpes or herpes proctitis and was later diagnosed with mpox 5 days after first healthcare attendance.

### Case definitions

Mpox is a statutory notifiable disease in Northern Ireland [4]; clinicians are therefore required to notify PHA of suspected or confirmed cases. Definitions used to support case assessment, notification and confirmation were adapted from UK Health Security Agency (UKHSA) guidance [5]. This report includes all laboratory-confirmed mpox cases known to PHA during the reporting period 6–30 June 2026 (as at 2 July 2026).

## Public health response

On 8 June 2026, the PHA convened a core multi-disciplinary group, followed by a wider incident management team meeting on 11 June 2026. A standardised questionnaire was completed for each case by telephone. Cases were advised to self-isolate, and their contacts were risk-stratified and offered post-exposure prophylactic vaccination in accordance with UKHSA guidance [6].

An email alert was sent to primary and secondary care professionals, advising of the increase in mpox diagnoses in Northern Ireland. A further awareness session was held to raise awareness with emergency department, general practice and surgical professionals, given the pattern of case presentation to general and acute care settings. Public communications were also developed as part of the response to raise awareness of mpox symptoms and of vaccine eligibility in at-risk populations. The PHA worked with a lesbian, gay, bisexual, queer plus (LGBTQ+) voluntary sector organisation to increase the reach of public messaging, including through promotion of mpox vaccine on targeted dating applications.

## Laboratory testing and sequencing

Mpox diagnostic testing was performed at the Regional Virology Laboratory in Belfast using a real-time PCR test for monkeypox virus (MPXV) detection [7]. Clade discrimination was followed up using real-time PCR assays for clade Ia, Ib and II, which target the complement-binding gene (C3L) and tumour necrosis factor receptor gene [7,8].

Specimens from the first three confirmed cases were referred to the Rare and Imported Pathogens Laboratory at the UKHSA for whole genome sequencing. Preliminary analysis of consensus sequences using Nextclade confirmed clade Ib infection in all three cases. The sequences fell within a wider cluster containing genomes from the ongoing clade Ib mpox outbreak in Europe 2026. Sequencing of further outbreak specimens is still ongoing.

## Discussion

We describe an ongoing outbreak of clade Ib mpox in Northern Ireland, which is the first detection of clade Ib infection in this jurisdiction. Before June 2026, there had been no detection of mpox infection in Northern Ireland for over 12 months, and, within this same period, notification of clinically suspected cases had been relatively infrequent. In the first month of this outbreak, Northern Ireland recorded the highest monthly total of clade Ib mpox confirmed cases of the four nations in the UK [2]. In the context of recent UK case numbers [2], the size of this outbreak (22 confirmed cases) is particularly notable, given the relatively small population size of Northern Ireland [9].

Risk communication during this response raised several ethical considerations, including the balance between undertaking public health actions (e.g. contact tracing), the need for public awareness and transparency, and the duty to protect confidentiality, particularly within a small population. While contact tracing informed the public health response, detailed findings are not presented herein due to of the risk of deductive disclosure of sensitive personal information. Throughout this response, safeguarding individual confidentiality was essential to maintaining trust and facilitating an effective public health response. Communications also had to be targeted to raise awareness without exacerbating stigma towards marginalised groups. An established partnership with an LGBTQ+ voluntary organisation was used to enable prompt co-development of inclusive and accessible communication materials.

The UK experience of the 2022 mpox incident was that cases presented primarily to specialist SHH services [10,11]. This may have been facilitated by the considerable public health and media communication activity surrounding the 2022 global outbreak. In our current outbreak, by contrast, 10 of 22 cases presented to emergency departments or out-of-hours care. In eight of these 10 cases, mpox was not tested for at the initial healthcare appointment.

This outbreak has been characterised by severe anorectal disease and a higher proportion of hospitalised cases compared with that observed in the previous 12 months in the European Union/European Economic Area [12]. Almost a quarter of cases required hospital admission (5/22), which was higher than reported in international and UK case series [11,13,14]. Rather than the emergence of a new, more virulent strain of MPXV, this may reflect early outbreak ascertainment bias, given the genomic similarity of the isolates to strains circulating in the UK and Europe.

Anorectal disease, including proctitis, is a recognised feature of mpox [15] and has been associated with hospitalisation [16,17]. Proctitis was common in this outbreak, consistent with a reported pooled prevalence of 25% [17]. Mpox-related proctitis may occur with or without limited typical mucocutaneous lesions [17,18]. In several cases in this outbreak, localised genital or peri-anal disease preceded, or occurred without, a prominent generalised rash. The low initial lesion burden in some cases here may have contributed to delayed recognition, particularly among individuals presenting to settings outside of SHH services. The need to extend case recognition beyond SHH services to emergency and surgical specialties, including for patients without typical rash, has been described previously [19]. As clade Ib mpox continues to spread in the UK and Europe, these findings highlight the need for awareness of anorectal mpox presentations, including anogenital lesions and/or proctitis without typical rash.

## Conclusion

The early management of this clade Ib mpox outbreak has been complicated by presentation of several cases with symptoms not recognised as a sexually transmitted infection. Cases have presented to settings outside of SHH services and have been hospitalised, with some requiring surgical intervention for proctitis. Our experience demonstrates the potential for a rapid growth in case numbers over a short time period and emphasises the need for increased public awareness of the ongoing risks of acquiring mpox as a sexually transmitted infection in Europe, particularly during the summer months, when travel-associated infections are more likely. Continued promotion of pre-exposure vaccination remains important for groups at highest risk of mpox, including gbMSM.

## Data Availability

Case level data will not be shared to protect the privacy of individuals. Genomic sequencing and analysis undertaken as part of this investigation represent preliminary work conducted by the UK Health Security Agency (UKHSA). The sequence data are not currently publicly available. Following validation, accession numbers will be added to this article as an Addendum, when available.
